# A Wideband Circularly Polarized Magnetoelectric Dipole Antenna for 5G Millimeter-Wave Communications

**DOI:** 10.3390/s22062338

**Published:** 2022-03-17

**Authors:** Hussain Askari, Niamat Hussain, Md. Abu Sufian, Sang Min Lee, Nam Kim

**Affiliations:** 1 Department of Information and Communication, Chungbuk National University, Cheongju 28644, Korea; askarihussain01@gmail.com (H.A.); abu.sufian@chungbuk.ac.kr (M.A.S.); 2 Department of Smart Device Engineering, Sejong University, Seoul 05006, Korea; niamathussain@sejong.ac.kr; 3 Department of Corporate Support Centre, Korea National University of Transportation, Chungju-si 27469, Korea; leesm@ut.ac.kr

**Keywords:** magnetoelectric, dipole, circularly polarized, 5G antenna, 28 GHz

## Abstract

In this paper, a wideband circularly polarized (CP) magnetoelectric (ME) dipole antenna operating at 28 GHz band was proposed for 5G millimeter-wave (mm-wave) communications. The antenna geometry included two metallic plates with extended hook-shaped strips at its principal diagonal position, and two corners of truncated metallic plates at the secondary diagonal position. The pair of metallic vias connected the modified strips to the ground plane to create the magnetic dipole. The L-shaped probe feed between the strips was used to excite the antenna. The antenna showed stable gain and wideband characteristics. The simulated and measured results showed that the proposed CP ME dipole antenna had an overlapping (|S_11_|< −10 dB impedance and 3 dB axial ratio) bandwidth of 18.1% (25–30 GHz), covering the frequency bands dedicated for 5G new radio communications. Moreover, an average gain of 8 dBic was achieved by the antenna throughout the operating bandwidth. The measured data verified the design concept, and the proposed antenna had a small footprint of 0.83 λ_o_ × 0.83 λ_o_ × 0.125 λ_o_ (λ_o_ is free space wavelength at the lowest operating frequency), suitable for its application in 5G smart devices and sensors.

## 1. Introduction

**Figure 1:**
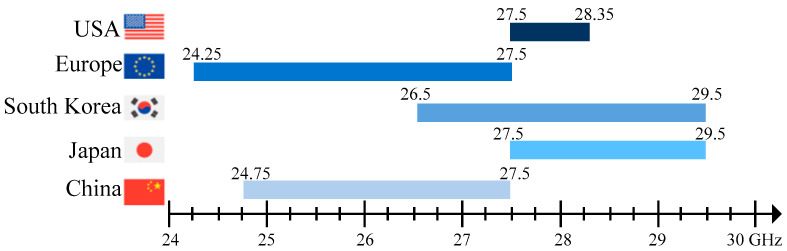
Global snapshot of the 5G millimeter-wave spectrum.

The twenty-first century has experienced tremendous development in the field of wireless communication. The fast evolving fifth-generation (5G) technology has brought new advancements which have posed a great challenge for antenna researchers and engineers. Large channel capacity and high data rates should be achieved to meet the demands of the 5G communication link. In this scenario, the millimeter-wave (mm-wave) band is used to fulfill the gigabit high-data transmission, and addresses the lack of wider spectral resource in the currently allocated band below 6 GHz [1]. Recently, the third-generation partnership project (3GPP) has allocated new radio (NR) frequency bands in the mm-wave range from 24.25 to 29.5 GHz, which are known as n257 and n258. Figure 1 shows the mm-wave frequency band distribution adopted by the leading 5G countries in the world [2]. 

In addition, circularly polarized (CP) antennas have been a unique choice for stable communication due to their resilience to multipath interference and polarization mismatching between receiving and transmitting antennas. These features make CP antennas the desirable candidate for numerous applications including satellite communication, radar communication, randomly oriented RFID (radio frequency identification) tags, GPS (global positioning system), and sensors [3,4,5,6,7,8,9]. Therefore, the design of CP antennas has always been a hot topic for antenna designers, compared with linear polarized (LP) antennas. In the literature, different kinds of CP antennas have been reported for mm-wave applications, including patch antenna, aperture antenna, slot antenna, cavity antenna, and magneto-electric (ME) dipole antenna. The ME dipole antenna is the most popular type of antenna due to its complementary performance.

The concept of the first ever complimentary antenna was proposed by Chlavin in 1954 [10]. Since then, many structures were proposed based on a slot/dipole or a slot/monopole combination. In 2006, Luk and Wong introduced a complementary antenna based on patch and dipole antenna combinations for the first time, achieving wideband performance [11]. The idea used a pair of copper patches parallel to the ground as an electric dipole and a pair of copper shorted patches perpendicular to the ground as a magnetic dipole. The antenna showed linear polarization with wide bandwidth, stable gain, and low levels of cross-polarization. In the course of designing ME dipole antennas [12,13], less attention has been paid to CP ME dipole based antennas. In the past few years, some CP antennas based on ME dipole structures have been reported [8,14,15,16,17,18,19,20,21]. In [14], an ME dipole structure was reported to use a pair of bowtie patch antennas and a pair of trapezoidal-shaped dipoles fed by a Wilkinson power divider to achieve a 3 dB AR bandwidth of 33%. Moreover, to ensure the CP radiation was presented, an ME dipole antenna was integrated with a crossed dipole fed by double phased delay rings in [15]. The proposed antenna achieved 27.67% of 3 dB AR bandwidth. In [16], substrate integrated waveguide (SIW) and aperture coupled feeding was used to excite an ME dipole antenna for mm-wave applications. This antenna achieved a 3 dB AR bandwidth of 12.8%. However, the above-mentioned reported ME dipole antennas showed limited AR bandwidth and had complex feeding structures. The wideband ME dipole antennas investigated in [17,18] had the advantage of a wider 3 dB AR bandwidth of 71.5% and 47.7%, respectively. However, the drawback of these antennas was their huge volume which limits their usage in modern electronic devices and sensors. On the other hand, the CP ME antennas designed at 60 GHz band offer wide AR bandwidth/high gain at the expense of large antenna size [19,20,21]. In [22], the AR bandwidth of the ME dipole antenna was significantly improved (53%) by employing a novel crossed feeding structure. This design has a high antenna profile (33.3 mm antenna height) and limited gain (6.6 dBic). A wideband circularly polarized ME dipole array fed by a complementary SIW power distribution phase shifter was presented [23]. The single-element antenna gain was 7dBic, while the AR bandwidth was restricted to 9.7%. To mitigate increased atmospheric losses in mm-wave bands, the gain of the wideband CP ME dipole antennas was increased in [24,25]. Moreover, the ME dipole antenna offered the advantages of low-profile and wide bandwidth with LP radiation [26]. In summary, the existing CP ME dipole antennas are suffering from either narrowband operation or high antenna profiles.

In this paper, a compact, wideband CP ME antenna operating at 28 GHz band is presented for 5G communications. The antenna’s CP bandwidth covers 25–30 GHz band (18.1%), covering the frequency bands dedicated for 5G new radio communications. Moreover, the antenna offers stable gain (average 8 dBic) and radiation patterns, with the advantages of a small footprint (0.9 λ_o_ × 0.9 λ_o_ × 0.14 λ_o_) for its applications in 5G smart devices and sensors. 

The rest of the manuscript has been structured as follows: Section 2 explains the detailed analysis and design of the proposed circularly polarized ME dipole antenna. The simulated and measured results have been presented in Section 3, while the paper has been concluded in Section 4. 

## 2. CP ME Antenna Design and Analysis

### 2.1. Antenna Geometry

**Figure 2:**
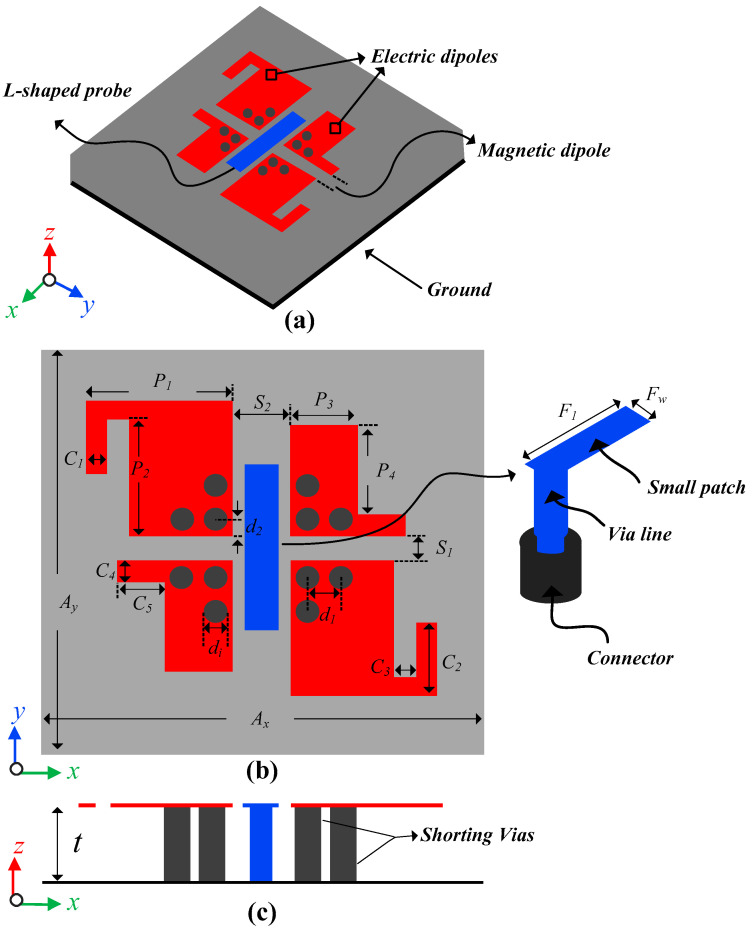
The geometry of the proposed CP ME antenna: (**a**) 3D view, (**b**) top view, and (**c**) side view.

The schematics of the proposed CP ME dipole antenna are shown in Figure 2. The antenna is composed of two metallic strips with an extended hook shape on its principal diagonal position, two L-shaped metallic strips on secondary diagonal position, and four sets of via holes. The modified metallic strips above the ground plane act as two planar electric dipoles. The four sets of via holes, each containing three metallic plated vias, are shorted between the modified patches and the ground plane. The metallic vias and the ground plane between them act as a magnetic dipole. The antenna is fed by an L-shaped probe to achieve low cross-polarization and back radiation levels [27,28]. The metallic patch of L-shaped probe acts as a coplanar waveguide (CPW) feed between the planar dipoles, which work as coplanar grounds. A square size (10 mm × 10 mm) Rogers 5880 with a thickness of 1.5 mm was used as the substrate of the antenna. The proposed ME dipole antenna’s optimized dimensions are summarized in Table 1.

During the antenna design, we found that the antenna offered optimum performance under the given constraints of the optimized parameters. Changing any parameter may disturb the AR or impedance bandwidth, or even both. In particular, varying the length of the patch (*F*_1_) changed the |S_11_| resonance of the antenna, however, the AR resonance did not change significantly. The distance between the monopole and metallic strips (*S*_2_) and *S*_1_ was sensitive to both |S_11_| and AR bandwidth. A small change may deteriorate the overlapping bandwidth. Similarly, all other parameters, especially *S*_1_, *C*_1_, *C*_2_, *C*_3_, *C*_4_, and *C*_5_ should be carefully tuned to achieve the best possible usable bandwidth.

### 2.2. CP Mechanism 

**Figure 3:**
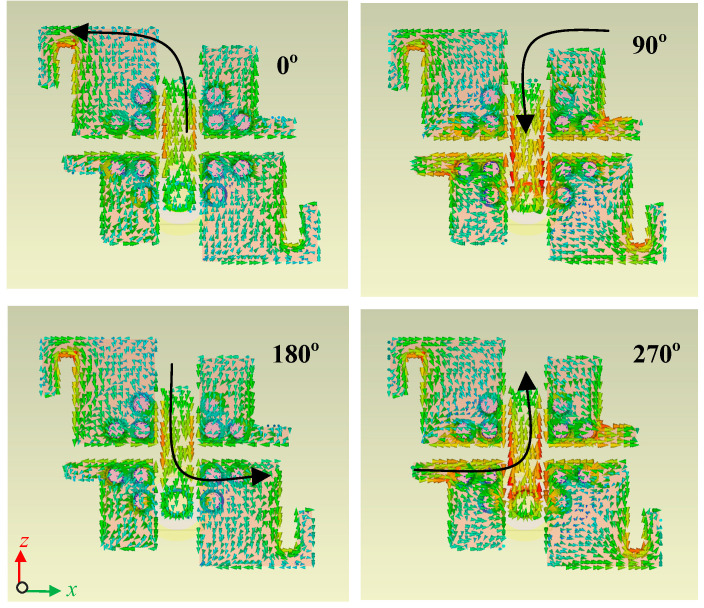
Surface current distributions of the proposed CP ME dipole antenna for different time phases at 28 GHz.

The CP mechanism of the proposed ME dipole antenna can easily be understood by visualizing the surface current distributions on coplanar electric dipoles, as shown in Figure 3. The surface current distributions were examined and recorded at 28 GHz for different time phases of 0°, 90°, 180°, and 270°. It can be noticed that the vector representation of surface current rotates in an anti-clockwise direction as the phase shifts from 0° to 90°, 180°, and 270°. The resultant vector of these surface current distributions makes a quasi-loop due to the rotational symmetry of this antenna, a necessary condition for CP radiation [29,30,31]. Thus, the proposed ME dipole antenna generates right-hand circular polarization (RHCP) in the positive *z*-axis direction.

The origin of CP radiations in the antenna is further explained by considering the degenerated modes which are excited in the planar electric dipoles and L-shaped microstrip patch antenna, separately. At time *t* = 0, the aperture of the shorted L-shaped patch acquires maximum surface current, and similarly for the time *t* = T/2, but at this latter time, the direction of surface current is opposite. Therefore, this causes induction of equivalent and orthogonal magnetic current in the aperture of the same L-shaped patch along the *x*-axis, which causes a 90 degree phase difference with respect to electric surface currents. In time *t* = T/4 and 3T/4, the planar electric dipoles attain maximum and opposite directed electric surface currents at their edges. It can be noted that the direction of electric surface currents on planar dipoles is also along the *x*-axis. Hence, the equivalent electric and magnetic currents are directed in the same direction and are in phase. This is how the proposed antenna can generate the CP radiation.

## 3. Results and Discussion

**Figure 4:**
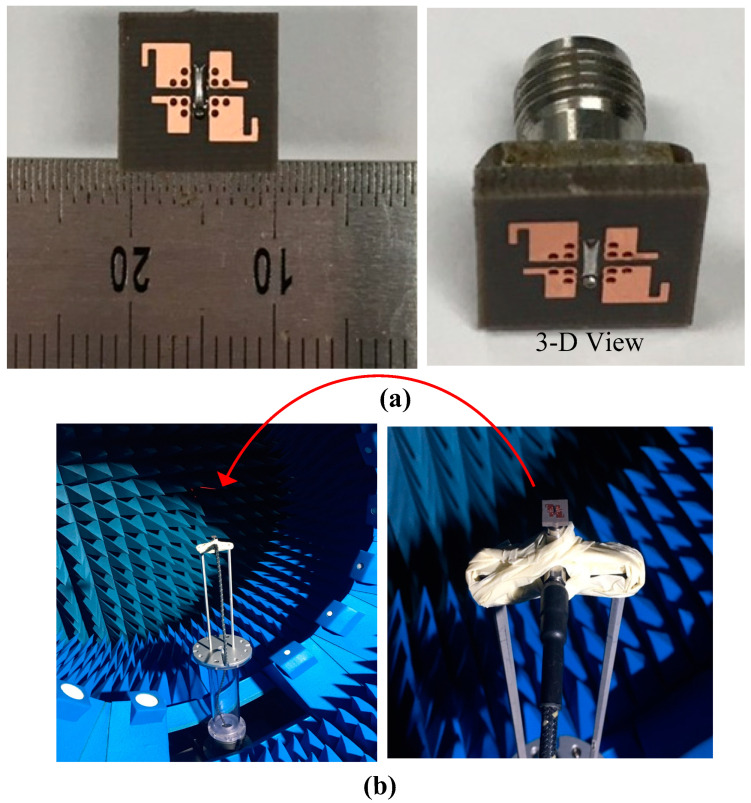
Photographs of the proposed CP ME dipole antenna: (**a**) fabricated prototype and (**b**) far field measurement setup.

The proposed CP ME dipole antenna’s prototype was fabricated and measured to verify its performance, as shown in Figure 4. Figure 4a shows the fabricated prototype of the proposed antenna. The setup for the antenna far field measurements is illustrated in Figure 4b. The antenna was measured in a multi-probe 360 degree scanning anechoic chamber. Overall, the simulated results showed good agreement with the measured results.

### 3.1. S-Parameters and Axial Ratio

**Figure 5:**
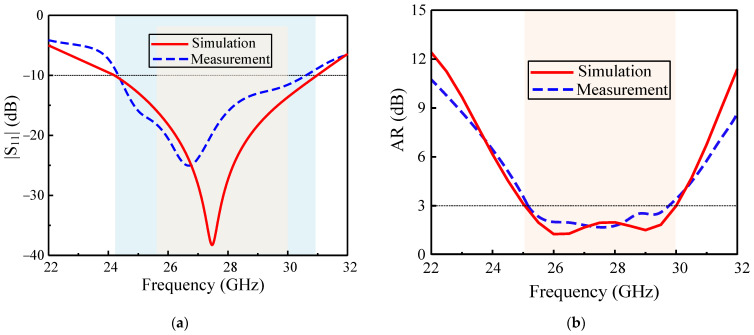
Proposed CP ME dipole antenna: (**a**) S-parameter |S_11_|and (**b**) axial ratio.

The measurement result for the S-parameter |S_11_| was obtained using a network analyzer (Rohde and Schwarz ZVA 40) in open air condition. The little difference in simulated and measured results, as shown in Figure 5a, was due to connector/cable losses. The proposed antenna has an impedance bandwidth for |S_11_| < −10 dB was 24.6%, that is, from 24.1 to 31.0 GHz. The simulated and measured 3 dB AR bandwidth of the proposed antenna was 18.1%, ranging from 25 to 30 GHz, as shown in Figure 5b. Thus, the overlapping operating bandwidth of the antenna covered the important band spectrum proposed for 5G mm-wave applications.

### 3.2. Broadside Gain and Efficiency 

**Figure 6:**
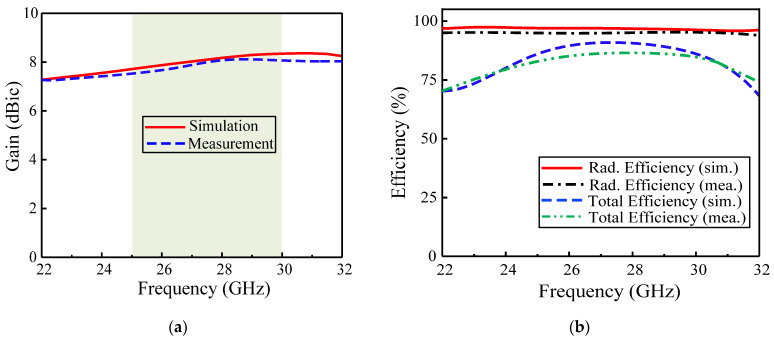
Proposed CP ME dipole antennas: (**a**) broadside gain, (**b**) radiation efficiency and total efficiency.

The simulated and measured broadside gain of the proposed antenna is shown in Figure 6a. The antenna showed a stable broadside gain of 8 dBic with a deviation of only ±0.5 dBic within the frequency range of interest. Moreover, the antenna also offered high efficiency, both total and radiation efficiency, due to good antenna impedance matching (Figure 6b). The simulated radiation efficiency of the antenna was more than 96%, which was in close agreement with its measured value. The measured total efficiency was observed to be a little lower than its simulated values (88%) due to possible cable/connector and substrate losses.

### 3.3. Radiation Patterns

**Figure 7:**
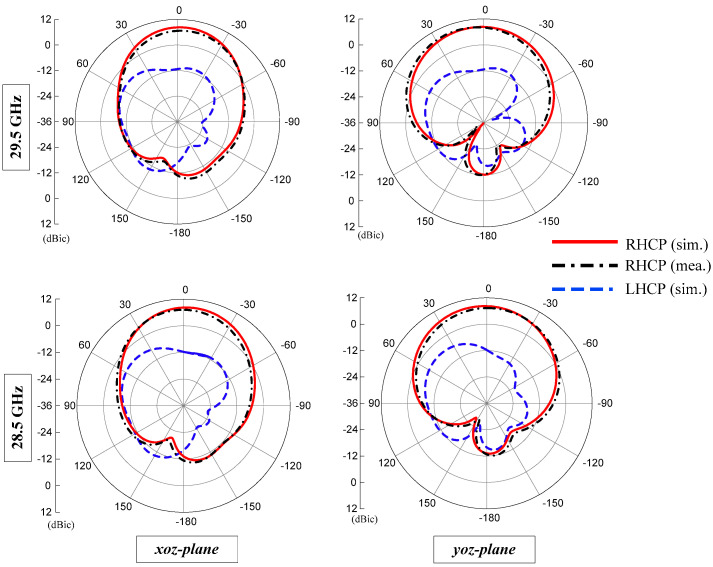
Radiation patterns of the proposed CP ME dipole antenna for different frequencies.

The measured and simulated radiation patterns of the proposed antenna are shown in Figure 7. The radiations patterns were analyzed in both the *xz*- and *yz-*principal planes for 28.5 GHz and 29.5 GHz frequencies. Since the LHCP radiations were minor, the antenna had stable RHCP radiation patterns. At 28.5 GHz, the antenna showed a gain of 8.2 dBic, a front to back ratio of 26.9 dB, and an angular beamwidth (3 dB) of 61.1 and 74.3 in *xz*- and *yz*- planes, respectively. At 29.5 GHz, the antenna showed a gain of 8.3 dBic, a front to back ratio of 26.7 dB, and angular beamwidth (3 dB) of 58 and 76.5 in *xz*- and *yz*- planes, respectively.

### 3.4. Performance Comparison 

Table 2 shows the comparison of the proposed antenna with the similar state-of-the-art CP ME dipole antennas. To ensure a fair comparison, different performance metrics including antenna design geometry, antenna volume, frequency of operation, polarization, AR bandwidths, and the peak gain value are considered. It can be seen from the table that the proposed antenna demonstrated outstanding performance in terms of overall size, AR bandwidth, and peak gain. Many interesting designs of CP antennas based on ME dipole structure have been reported [8,16,17,18,19,20,21,22,23,24,25]. The proposed antenna is the smallest among its competitors with comparable gain and CP bandwidth. The antennas presented in [8,16] have smaller gain narrow bandwidth. The antennas presented in [17,18,19] have superiority in terms of bandwidth at the expense of larger antenna size, although, their gain performance is comparable with our proposed antenna. The antenna design in [20] has the advantages of higher gain (10.4 dBi), however, it has the limitations of narrower bandwidth and larger antenna profile. Similarly, the mm-wave ME CP antenna in [21] has the merits of wide bandwidth but has lower gain and a larger size. Although the design developed in [22] offers a very wide AR bandwidth due to its novel feeding mechanism, it has limited gain and 3D geometry with a high antenna profile. The CP ME antenna arrays offer wideband and high gain due to their increased number of radiating elements, which, of course, increases the antenna size and complex feeding networks [23,24,25]. It is noted that our antenna consists of a single element and has a stable gain (average 8 dBic) with a deviation of only ±0.5 dBic. In conclusion, the proposed antenna outperforms the existing CP ME dipole antennas with its highest gain of 8.5 dBic, wide 3 dB AR bandwidth (18.1%), and a small volume of only 0.83 λ_o_ × 0.83 λ_o_ × 0.125 λ_o._ Since MIMO antennas with increased gain are the key requirements for the 5G systems [32], this work can be extended for MIMO configuration with enhanced isolation in the future.

## 4. Conclusions

An ME dipole antenna with RHCP radiation for unidirectional radiation characteristics is characterized and achieved for 5G mm-wave communication systems. The proposed antenna comprises two pairs of rotational symmetric electric dipoles and two pairs of metallic vias perpendicular to the ground plane, acting as a magnetic dipole. An L-shape probe is used to excite the antenna. Owing to the shape of the electric dipoles together with the complementary effect, it produces CP radiations. This antenna has impedance bandwidth of 24.6 for |S_11_| < −10 dB and 3 dB AR bandwidth of 18.1%. The antenna has achieved a peak gain of 8.5 dBic with a compact overall size of 0.83 λ_o_ × 0.83 λ_o_ × 0.125 λ_o_. The wide CP bandwidth, stable radiation characteristics, high efficiency, and low profile of the proposed antenna makes it a suitable candidate for 5G smart devices and sensors.

## Figures and Tables

**Table 1:** Optimized dimensions of the proposed CP ME dipole antenna.

Parameter	Value (mm)	Parameter	Value (mm)	Parameter	Value (mm)
*t*	1.5	*P*_4_	1.8	*C*_4_	0.45
*F*_1_	2.5	*S*_1_	0.5	*C*_5_	1
*F_w_*	0.7	*S*_2_	1	*d_i_*	0.5
*P*_1_	3	*C*_1_	0.4	*d*_1_	0.7
*P*_2_	2.4	*C*_2_	1.5	*d*_2_	0.35
*P*_3_	1.4	*C*_3_	0.5	*A_x_*, *A_y_*	10

**Table 2:** Performance comparison of the proposed CP ME dipole antenna with the reported works.

Ref.	Antenna Type	Antenna Volume (λ_0_^3^)	*f_c_* (GHz)	Polarization	3 dB ARBW (%)	Peak Gain (dBic)
[8]	ME dipole antenna	1.54 × 0.48 × 0.291	29	CP	4.8	5.1
[16]	ME dipole antenna	1.18 × 1.05 × 0.122	24	CP	12.8	7.8
[17]	ME dipole antenna	1.6 × 1.3 × 0.26	2.5	CP	71.5	8
[18]	ME dipole antenna	1.1 × 1.1 × 0.29	2.2	CP	47.7	8.6
[19]	ME dipole antenna	6 × 6 × 0.305	60	CP	23.4	8.6
[20]	ME dipole antenna	1.12 × 3.03 × 0.6	60	CP	11.6	10.4
[21]	ME dipole antenna	1 × 1 × 0.315	60	CP	21.9	7.9
[22]	ME dipole antenna	0.85 × 0.85 × 0.18	2.3	CP	53.2	6.6
[23]	ME dipole antenna array	Not given	28.8	CP	9.7	7
[24]	ME dipole antenna array	Not given	35.2	CP	44	19.2
[25]	ME dipole antenna array	Not given	27	CP	27.8	20.2
This work	ME dipole antenna	0.83 × 0.83 × 0.125	28	CP	18.1	8.5

## Data Availability

All data are included in the manuscript.
